# Supplementary figures and images for: Antigenic variation is caused by long plasmid segment conversion in a hard tick-borne relapsing fever Borrelia miyamotoi
Source: PLoS Pathog. 2025 Sep 30;21(9):e1013514. doi: 10.1371/journal.ppat.1013514 (PMC12510640; doi:10.1371/journal.ppat.1013514)

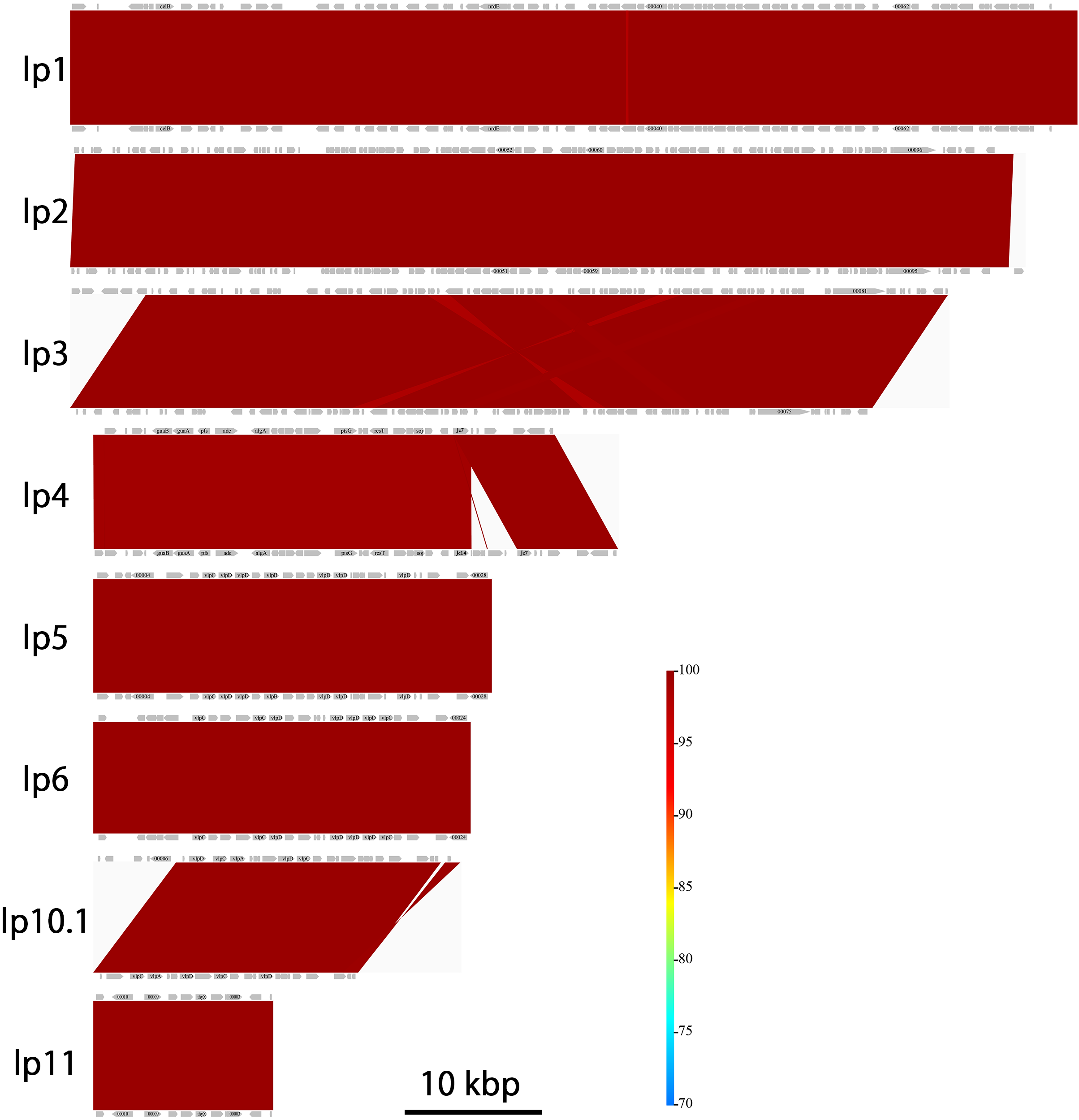

Supplement: S1 Fig — The comparison of plasmid size and gene synteny of B. miyamotoi MYK1 G3 and M1-2Br H4 were shown using GenomeMactcher3.10. The high similarity value is shown in red and only results with homology >98% are shown in the figure. Upper and Lower indicate the MYK1 G3 and M1-2Br H4, respectively. A 4,593-bp relative deletion was found in lp4. In the MYK1 G3 strain, from Jc14 locus to upstream of Jc7 locus in the M1-2Br H4 strain was missing. The sequence of Jc7 and Jc14 was conserved at the first 180 bp of 5′ region, while the 3′ region was highly variable. (TIF) [file ppat.1013514.s001.tif]

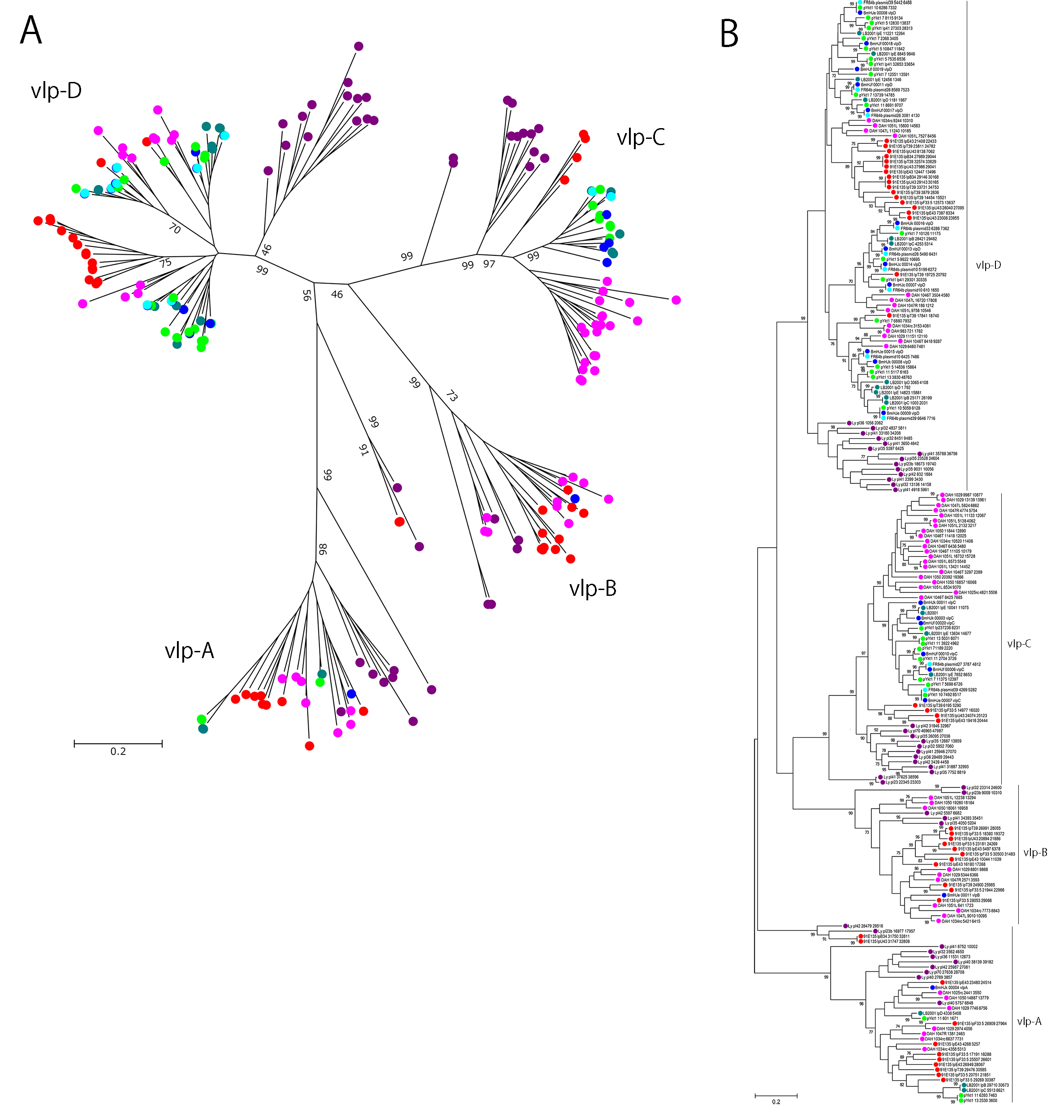

Supplement: S2 Fig — The unrooted (A) and rooted (B) Maximum Likelihood tree based on the Kimura 2-parameter model was constructed using sequences of vlp of soft-tick borne RF borreliae; B. duttonii Ly, B. turicatae 91E135 and B. hermsii DAH, indicated by purple, red and pink, respectively, and hard-tick borne RF borreliae; B. miyamotoi Yekat-1, LB-2001, FR64b and M1-2Br H4 indicated by light green, dark green, light blue and blue, respectively. The reference sequences were downloaded from GenBank database. (TIF) [file ppat.1013514.s002.tif]

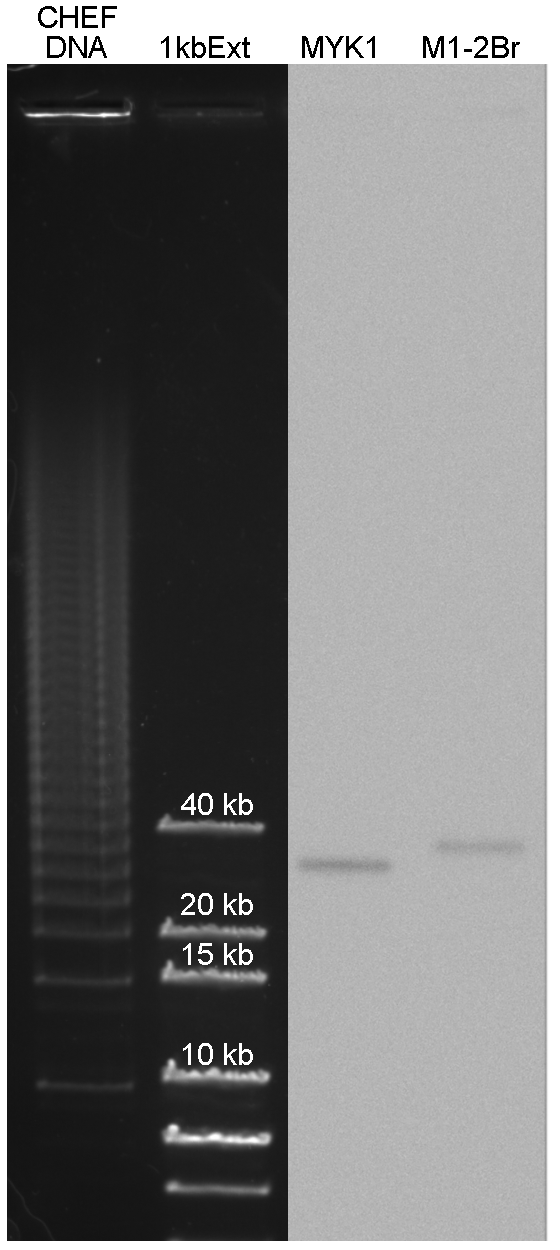

Supplement: S3 Fig — The southern blot analysis using a promoter sequence-specific probe for B. miyamotoi MYK1 G3 and M1-2Br H4 were shown. The PFGE gel was used for southern blot analysis of lp4. The promoter region of vmp locate at lp4 was labeled. This region was encoded on a single plasmid and the size difference between two strains was consistent with the results of the genome analysis. Molecular size markers that are denoted on panel in kilobase pairs. (TIF) [file ppat.1013514.s003.tif]

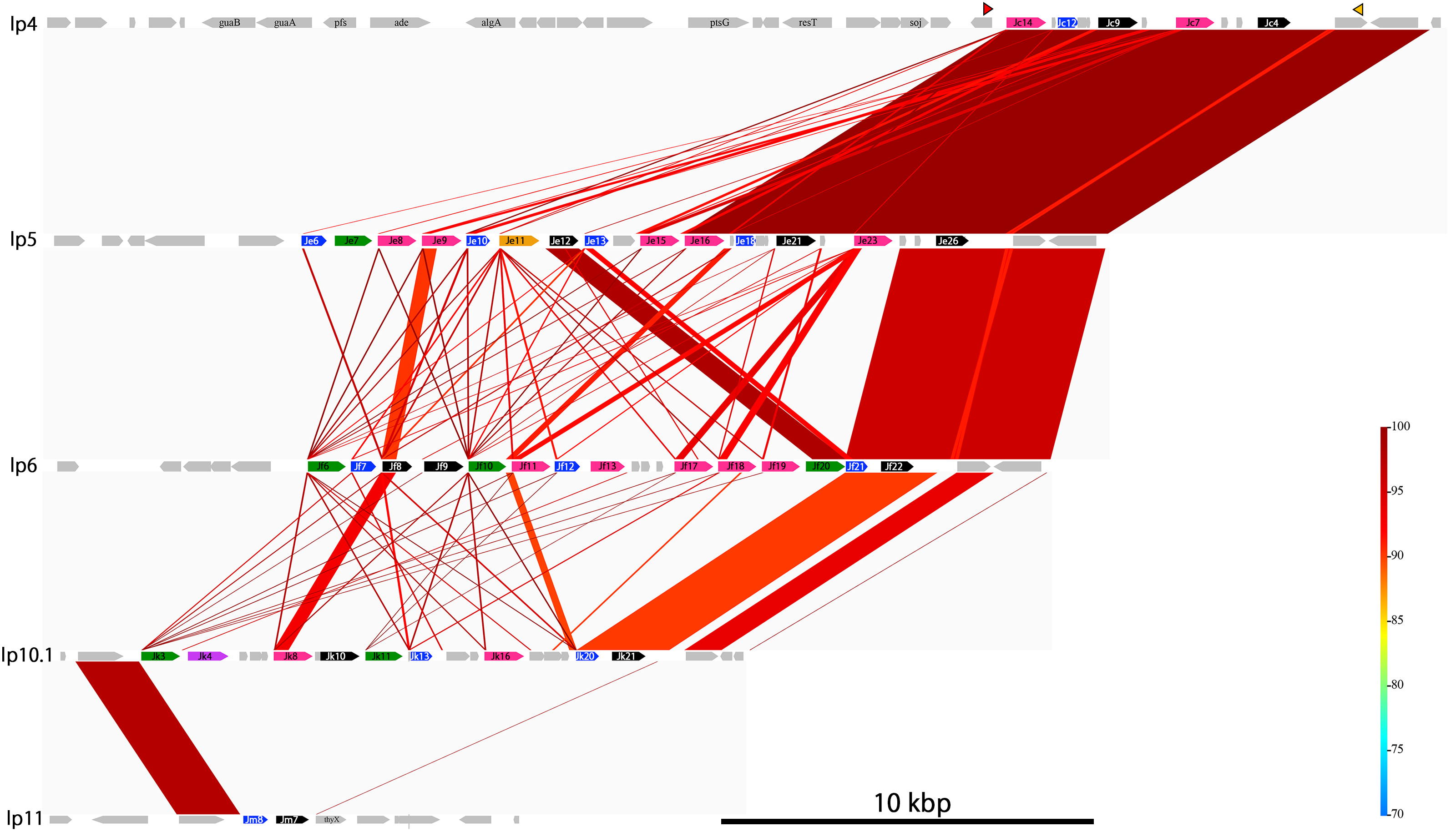

Supplement: S4 Fig — The 5 plasmids, lp4, lp5, lp6, lp10.1, and lp11, which vmp cassette-bearing plasmids were compared using GenomeMactcher3.10. The high similarity region was detected in the right end sequences of lp4, lp5, lp6, and lp10.1. The high similarity value is shown in red and only results with homology >90% are shown in the figure. Red and yellow arrowhead indicates the primer sites on lp4 of Vmp-UHR-F and Vmp-LR, respectively. (TIF) [file ppat.1013514.s004.tif]

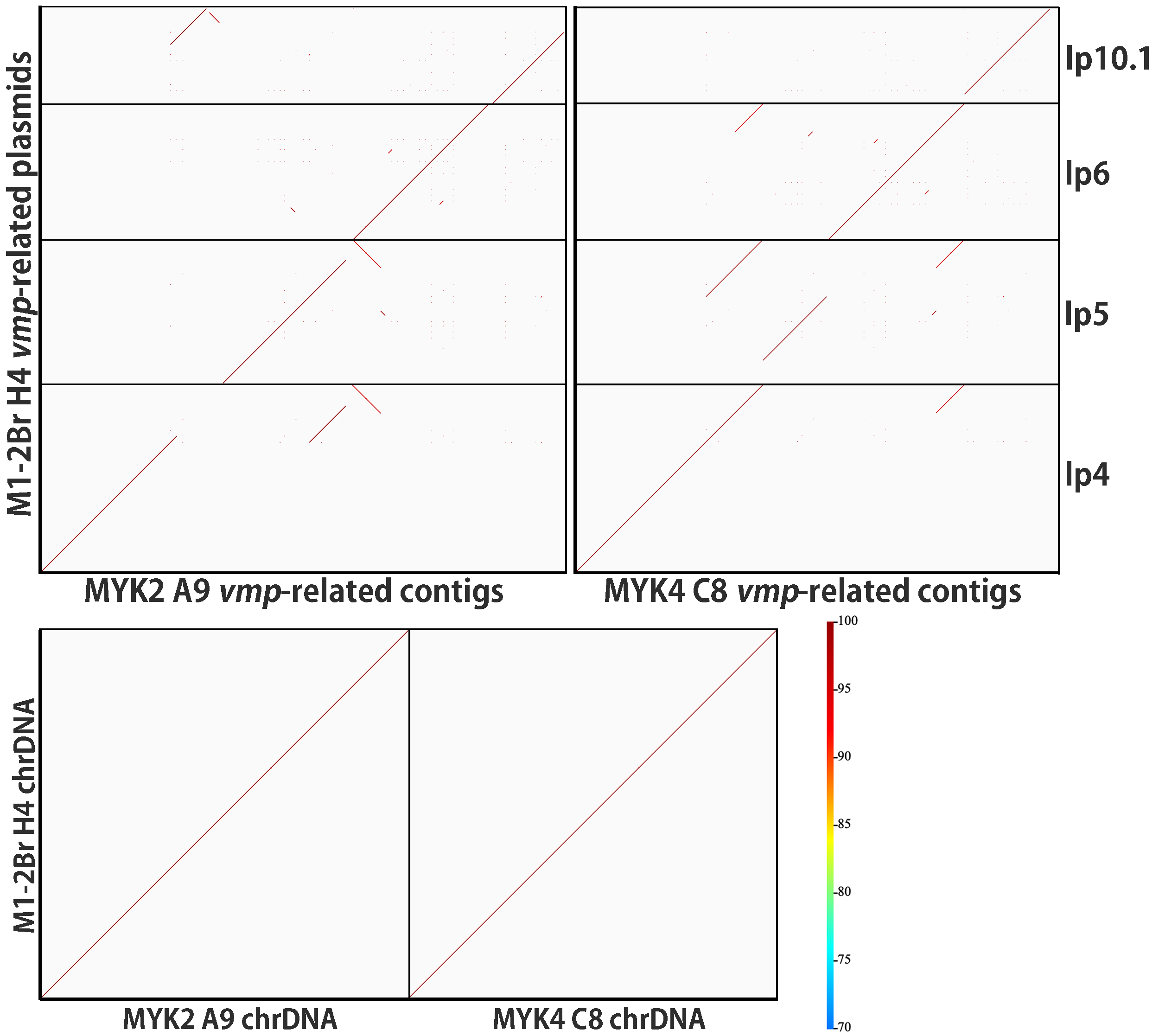

Supplement: S5 Fig — The chrDNA and vmp cassette-bearing plasmids of three strains were compared using GenomeMactcher3.10. The high similarity value is shown in red and only results with homology >95% are shown in the figure. Although the plasmid sequence of MYK2 A9 and MYK4 C8 were not verified in detail, the contigs carrying the silent cassettes were selected for comparison. It was suggested the gene repertoire of silent cassettes was conserved in three strains. (TIF) [file ppat.1013514.s005.tif]

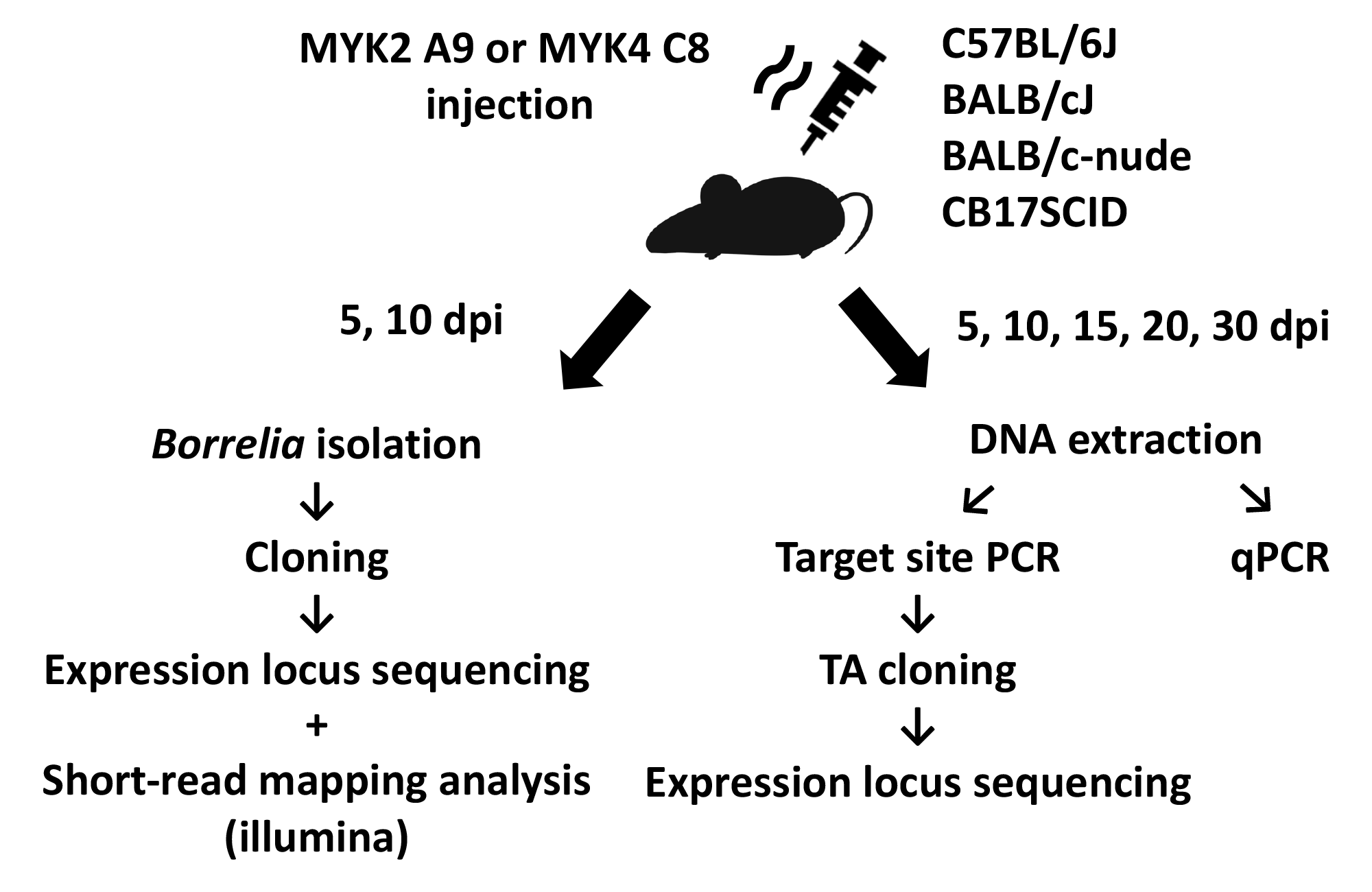

Supplement: S6 Fig — The schema of mice experiments was shown. The clipart of mice in this figure was downloaded from open source resources (URL: https://www.ac-illust.com/). (TIF) [file ppat.1013514.s006.tif]

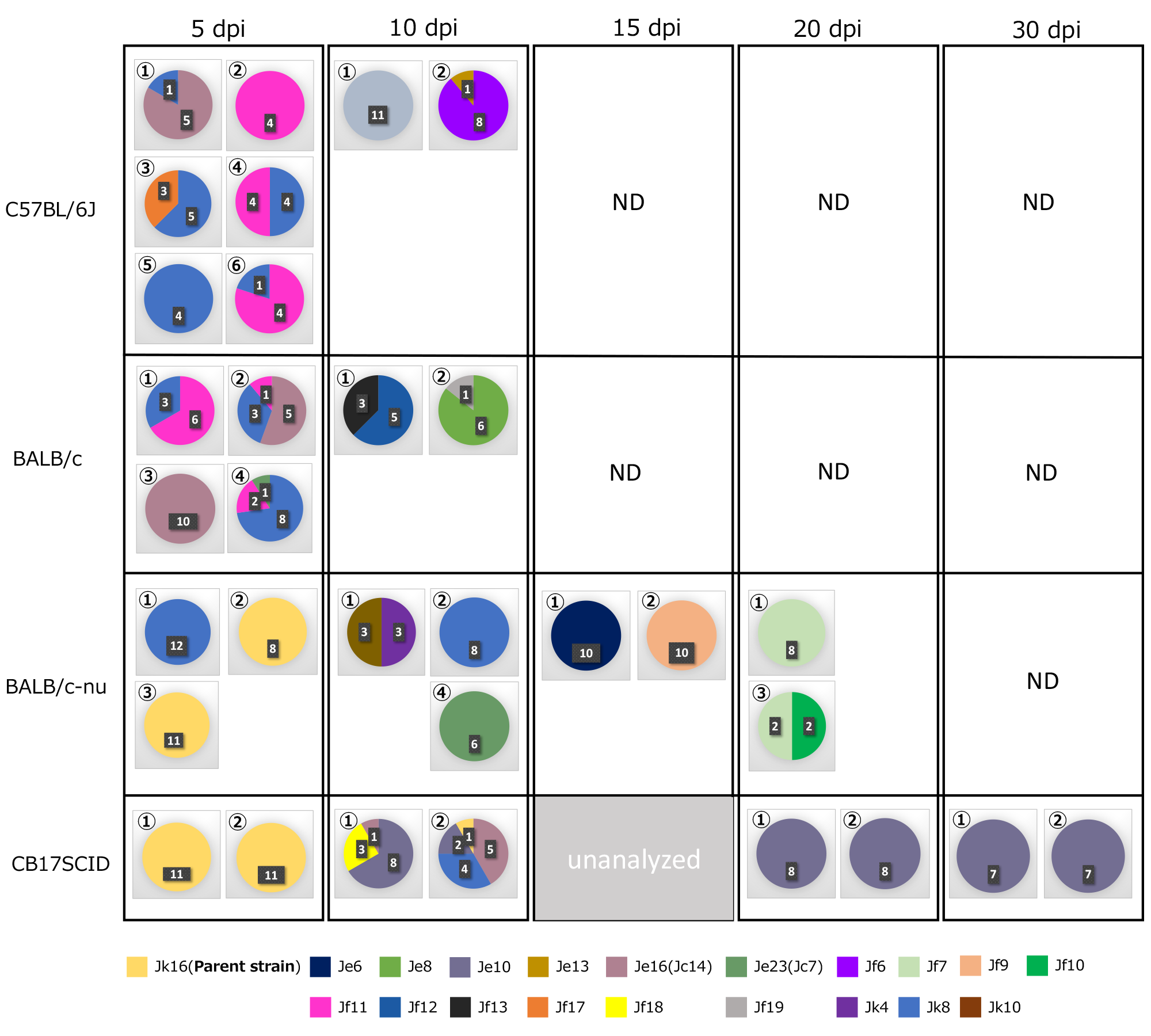

Supplement: S7 Fig — The frequencies of expression cassettes were analyzed by TA cloning. The parental strain expressed Jk16 and the change in expression vmp genes was observed from 5 dpi in immunocompetent mice. The circled number indicate individual mouse and the number in the pie charts is the number of clones detected from a mouse. ND: Not detected. (TIF) [file ppat.1013514.s007.tif]

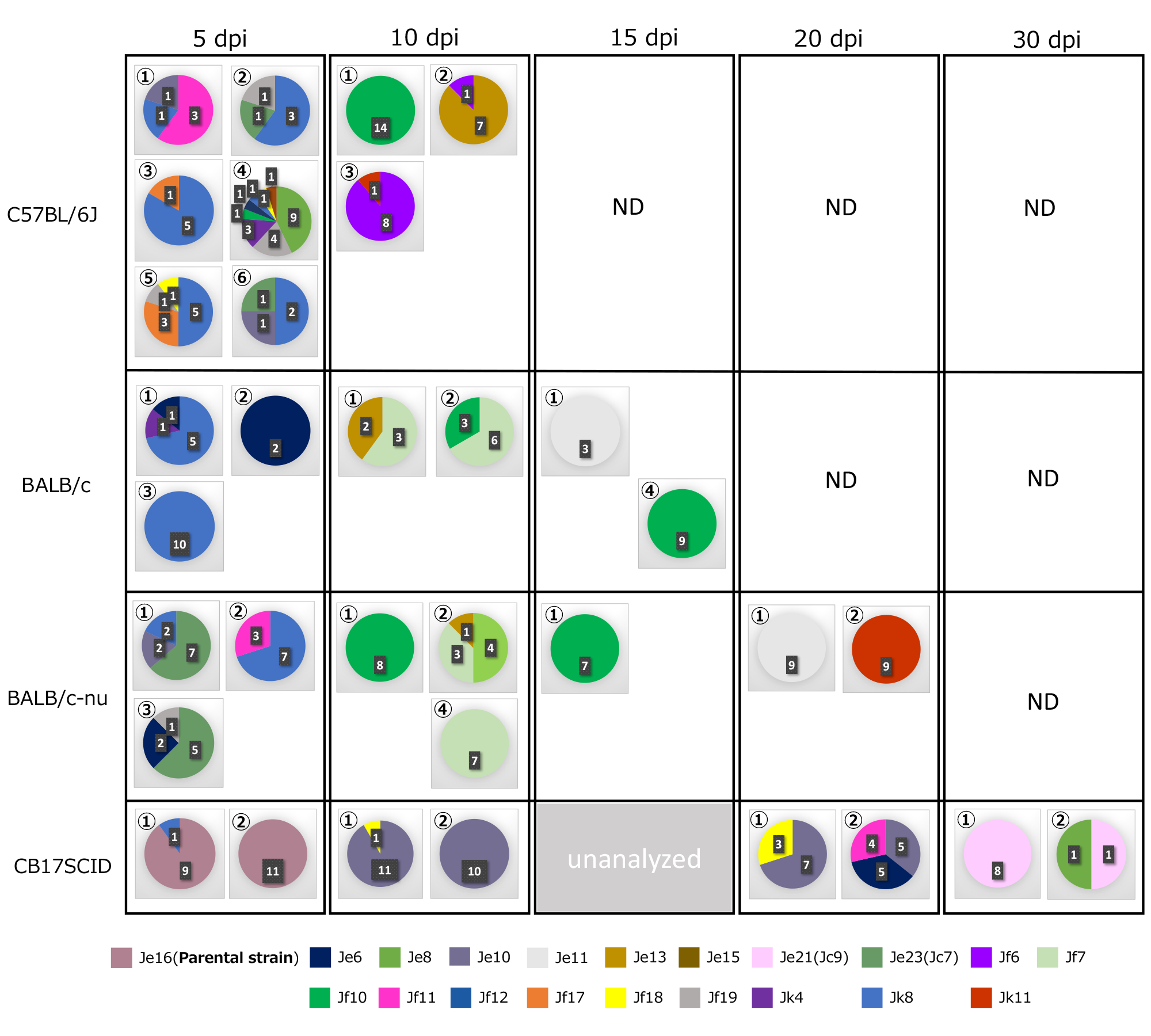

Supplement: S8 Fig — The frequencies of expression cassettes were analyzed by TA cloning. The parental strain expressed Je16 and the change in expression vmp genes was observed from 5 dpi in immunocompetent mice. The circled number indicate individual mouse and the number in the pie charts is the number of clones detected from a mouse. ND: Not detected. (TIF) [file ppat.1013514.s008.tif]

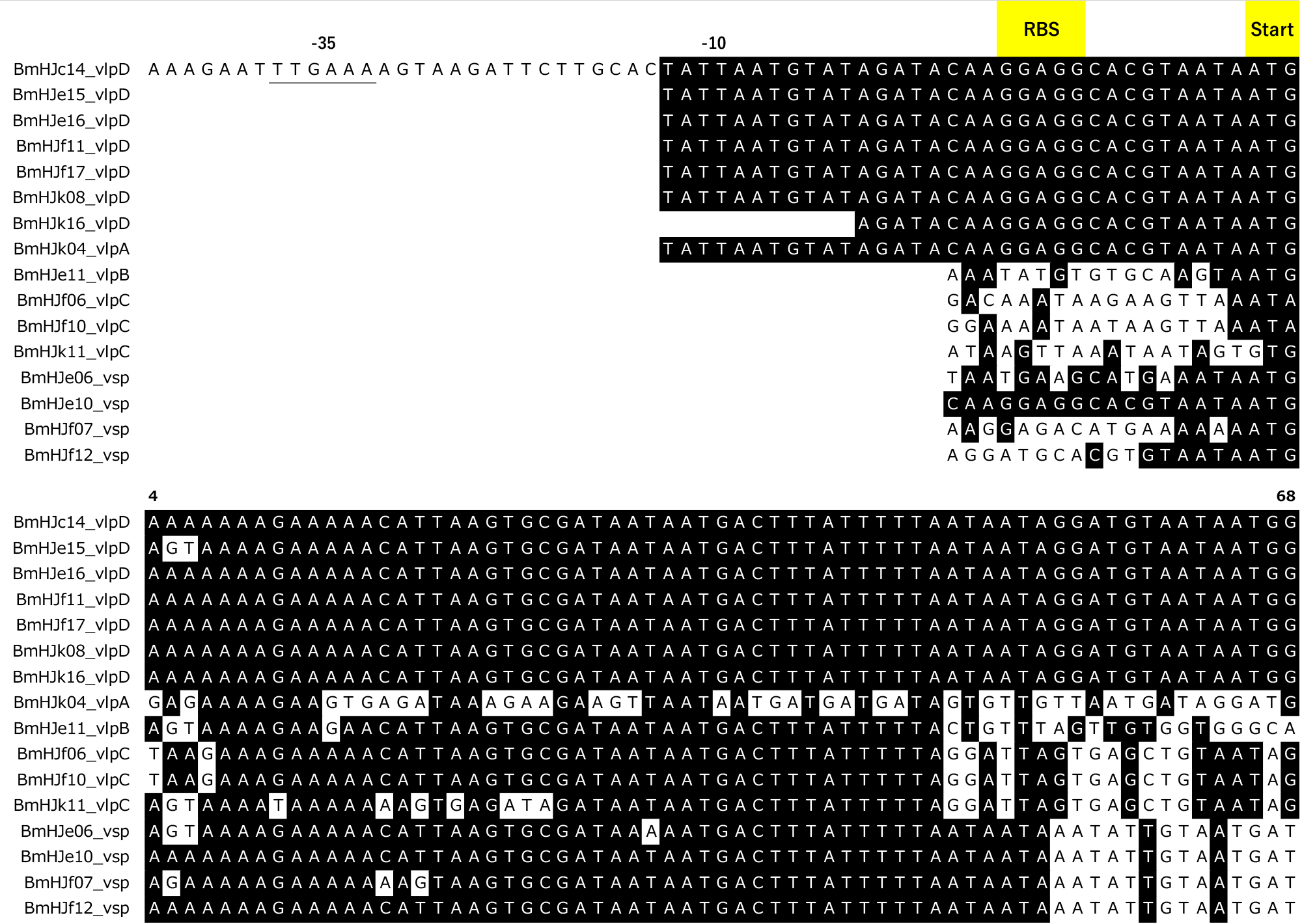

Supplement: S9 Fig — The UHS of the vmp expression locus and each silent cassette on M1-2Br H4 plasmid sequencing were compared by in silico analysis. RBS; ribosome binding locus. Start; start codon. Conserved nucleotides are shown on a black background. (TIF) [file ppat.1013514.s009.tif]

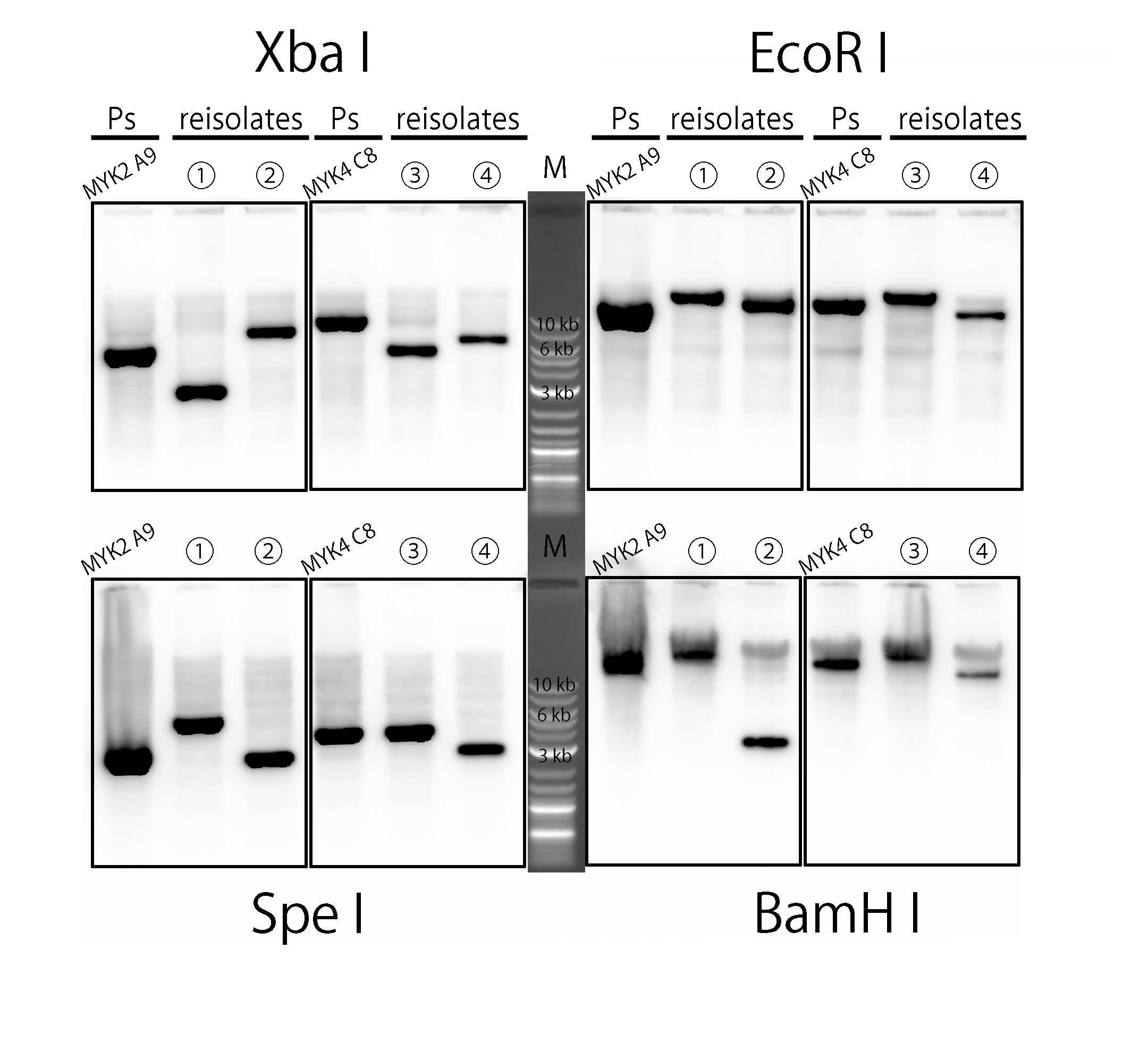

Supplement: S10 Fig — The southern blot analysis of B. miyamotoi parental strain and the reisolates was shown. The 4 restriction enzymes were used and the promoter region of vmp gene was labeled. The ① and ② indicate the re-isolated strains at 5 dpi injected MYK2 A9; B6M-1w A3 and B6M-1w C2, respectively. The ③ and ④ indicate the re-isolated strains at 5 dpi injected MYK4 C8; B6M-9L H6 and B6M-11w B1, respectively. Molecular size marker is denoted on the center of the panel in kilobase pairs. (TIF) [file ppat.1013514.s010.tif]

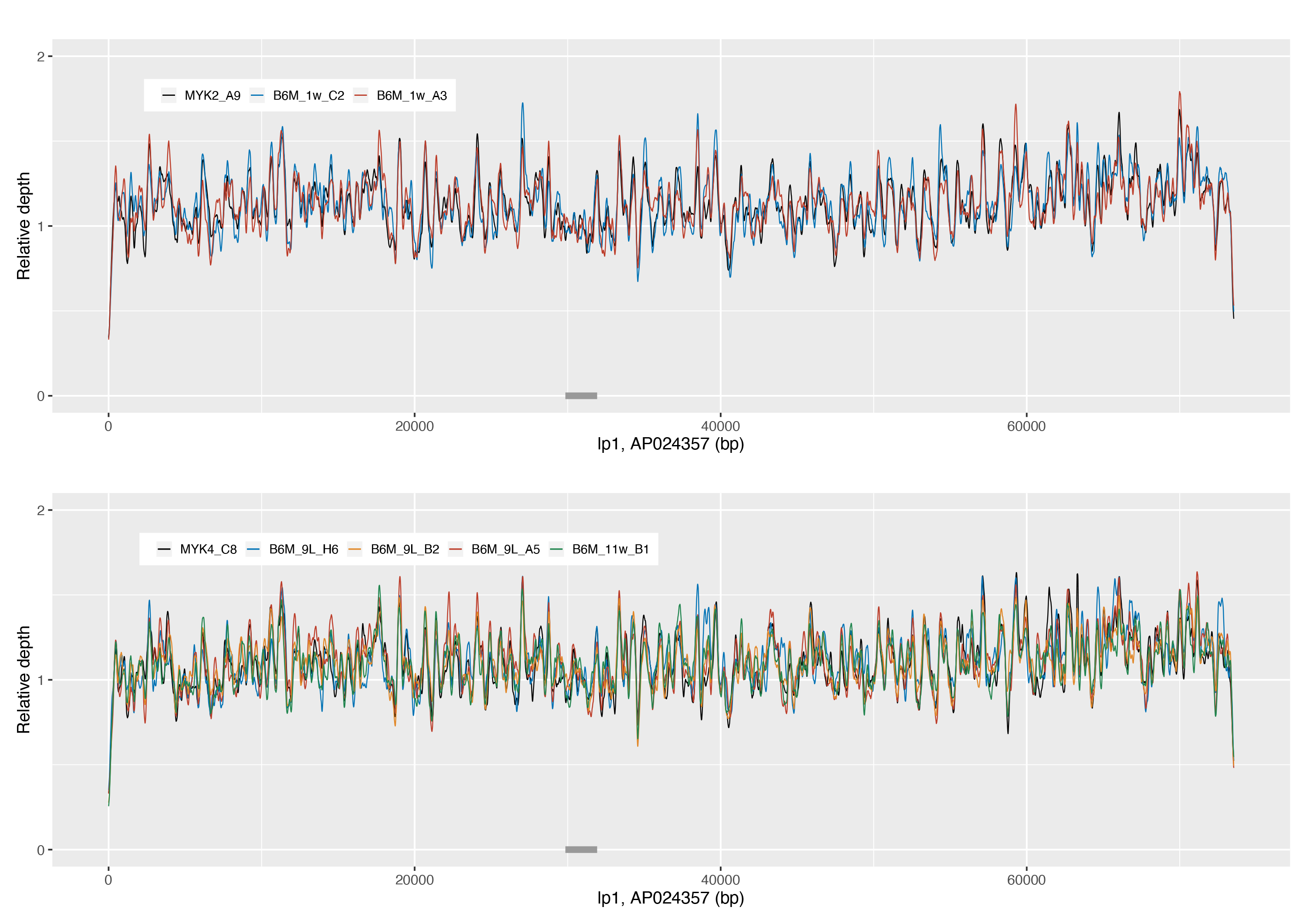

Supplement: S11 Fig — The Illumina reads of reisolates and their parental strains were mapped to the genome of M1-2Br H4. The expression locus segment of the M1-2Br H4 genome sequence (AP024359, 1–11967 bp) was masked and used as the reference. Mapping depth per base was calculated and normalized using the mean depth of the specified region (29852–31927, indicating the gray color). Mapping depths in 100-bp window sliding every 10 bp were depicted in R ver. 4.2.3 with ggplot2 package ver. 3.5.0. (TIF) [file ppat.1013514.s011.tif]

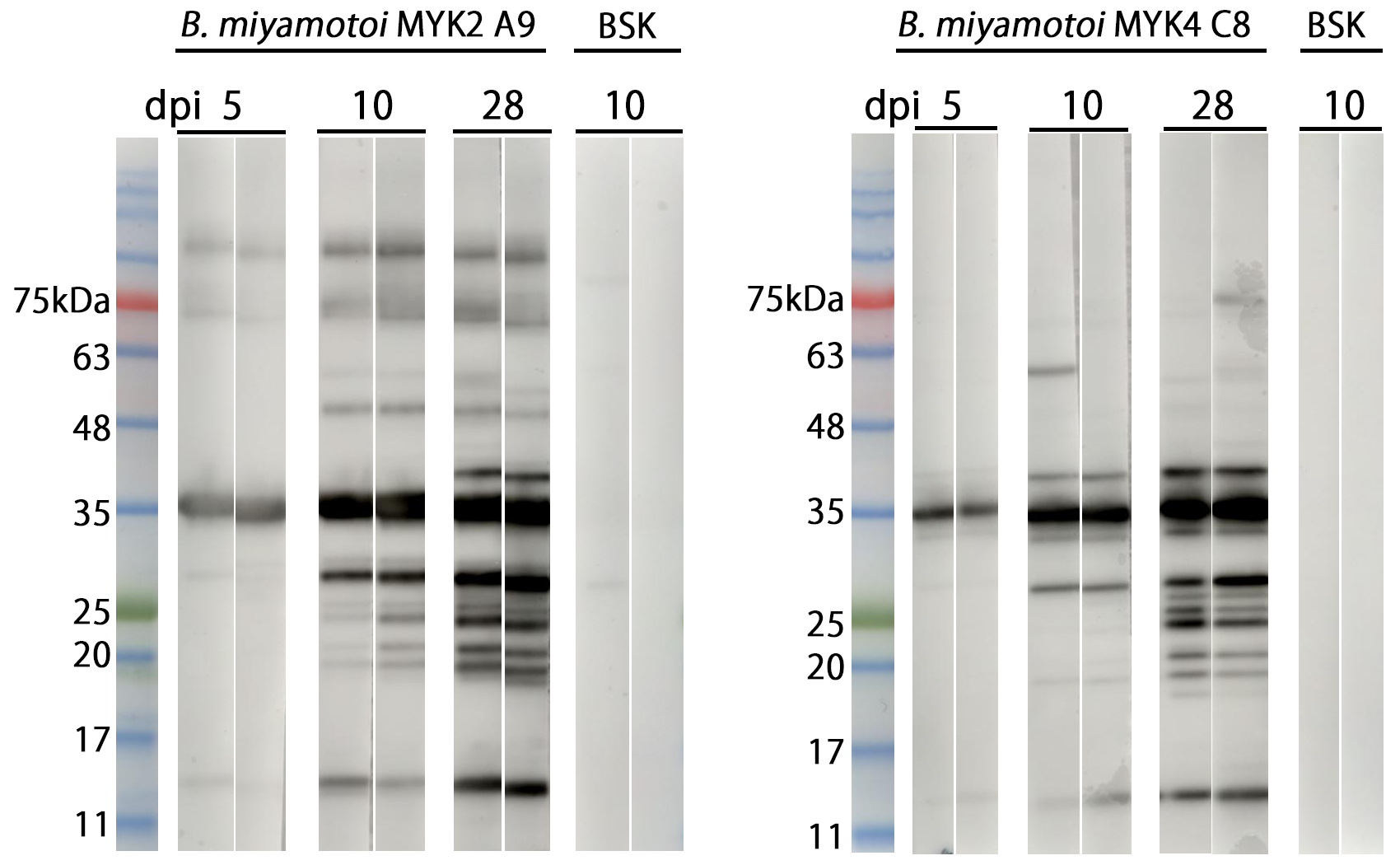

Supplement: S12 Fig — Seroconversion of mice inoculated with B. miyamotoi MYK2 clone A9 or MYK4 C8. Molecular mass is indicated on the left of molecular weight markers. Each lane showed the reaction of independent mouse serum collected at 5-, 10- and 28-days post-inoculation (dpi). The BSK medium inoculated mice serum collected at 10 dpi were used as control. (TIF) [file ppat.1013514.s012.tif]
