# Supplementary material for: Antigenic variation is caused by long plasmid segment conversion in a hard tick-borne relapsing fever Borrelia miyamotoi
Source: PLoS Pathog. 2025 Sep 30;21(9):e1013514. doi: 10.1371/journal.ppat.1013514 (PMC12510640; doi:10.1371/journal.ppat.1013514)
Supplement: Attachment — Submitted filename: Review of manuscript.pdf [file ppat.1013514.s020.pdf]

Review of manuscript "Antigenic variation is caused by long plasmid segment conversion in a hard tick-borne relapsing fever Borrelia miyamotoi"

General: This manuscript presents an interesting study that investigates a potentially novel mechanism of antigenic variation in Borrelia miyamotoi, involving long plasmid segment conversions. The authors combine genomic sequencing, mouse infection experiments, and molecular techniques to address this question. If confirmed, their findings could provide valuable insights into the biology of relapsing fever spirochetes. However, several methodological concerns and some overinterpretations of the data should be carefully addressed to ensure robustness and clarity before considering the manuscript for publication.

Minor comments:

Title: the title should

L64-65: To the best of my knowledge, *B. anserina* is the only RF species which transmitted by *Argas* ticks. If you mention it, the louse-borne species *B. recurrentis* should be mentioned as well.

L77-78: It is important to differentiate between different mechanisms. Trypanosomes antigenic variations is similar to *Borrelia* AV with gene conversion being the main driver, whereases other pathogens use different mechanisms such as point mutations, reassortment and epigenetic changes.

L110-112: Why is B. miyamotoi difficult to (re-)isolate from infected tissues? does this relate to low spirochetemia, fastidious growth requirements, or rapid antigenic shifts?

L120: which sequencing method was implemented?

L121-122: “None of the eight clones of MYK1, including clone G3, caused bacteremia when inoculated into laboratory mice (data not shown).”

What mouse strain was used? Inoculum conc.? Route of administration? I wonder whether intradermal vs. intraperitoneal inoculation alters early antigenic switching kinetics.

Did this fact affect sequencing method or results?

L124-126: Did the infected mice exhibit any clinical signs?

L127-133: read depth, assembly confidence around repetitive vmp regions and mapping coverage would strengthen claims of complete resolution.

L132-137: The 4.6 kb gap on lp4 is noted. Gap closure strategies like long-read polishing should be detailed to rule out assembly artifacts at the expression locus.

L141-142: What does “untypeable genes” mean? novel Vmp families or pseudogenes lacking hallmark motifs?

L220-232: Bacteremia dynamics are interpreted as evidence for antibody-mediated clearance. However, no direct measurement of antibody titers is provided. Correlating serology with bacterial decline is essential to link immune pressure and switching kinetics.

L291-292: Could this early conversion be classified as “spontaneous antigenic variation”, independent of adaptive immunity?

L306-314: The hypothesis of long-segment conversion assumes homology-driven recombination. However, the absence of DHS-like motifs suggests alternative recombination mediators. The authors should search for associated recombination-associated genes (encode resolvases or transposases) in proximity to vmp clusters.

L432-436: “The most important finding of this study was probably the elucidation of the mechanism underlying the change in expression *vmp* cassette (antigenic variation) in *B. miyamotoi”*. Although this study provides a comprehensive analysis of expression locus segment conversion in *B. miyamotoi,* the exact mechanism and conversion drivers remain unidentified.
