# Supplementary material for: Antigenic variation is caused by long plasmid segment conversion in a hard tick-borne relapsing fever Borrelia miyamotoi
Source: PLoS Pathog. 2025 Sep 30;21(9):e1013514. doi: 10.1371/journal.ppat.1013514 (PMC12510640; doi:10.1371/journal.ppat.1013514)
Supplement: Attachment — Submitted filename: Response to Reviewers.docx [file ppat.1013514.s022.docx]

Comments to the Authors

Reviewer 1

L. 127. It would be best here to remind the reader that MYK1 clone G3 and M1-2Br clone H4 were the clones sequenced.

Answer: Thank you for the comments. We modified as follows.

Lines 130-131: “By this sequencing, we obtained almost complete genome sequences of the two clones, MYK1 clone G3 (AP024375-AP024390) and M1-2Br clone H4 (AP024356-AP024370).”

L. 254. This paragraph is confusing and should be rewritten for clarity. In the text and Table S3, should be indicated what the initial expression vmp genotype was for each of the clones. For example, it cannot be assumed that all three clones of MYK2 A9 had Jk16. The paragraph as written simply restates what is in the table; perhaps it could be revised to provide a more general overview. It is surprising that none of the reisolated clones had the initial expression vmp still in the expression site (particularly at the 5 day time point). Was that the case? It seems that 5 days would be too short of a time period for the immune response to eliminate organisms expressing the initial expression vmp.

Answer: Thank you for the comments. We were also surprised that changes in expression *vmp* cassette occurred in such a short of a time, so we conducted multiple experiments. However, this phenomenon repeatedly observed at 5 days post-inoculation in multiple experiments. This result also confirmed by detection of expression *vmp* cassette in next experiment (Fig3). **Although spontaneous antigenic variation may occur at a very low frequency, factors other than antibodies may also contribute to this phenomenon. We have added a sentence to the Discussion section (L426).**

Please note that the *vmp* expression loci of these three reisolates B6M-1w, B6M-4L, and B6M10-22w of MYK2 A9 (Jk16) was not analyzed before cloning because it was impossible to determine the sequences of their *vmp* regions which had become heterogenous before cloning. Therefore, after cloning, we analyzed multiple clones of each reisolate by sequencing the *vmp* expression site and its downstream region. To clarify this schema of experiment, we have reconstructed this paragraph and cited Fig S6.

Lines 256-258: “The expression *vmp* cassette of these re-isolates had become heterogenous. Therefore, these re-isolates were cloned by limiting dilution, followed by analysis of expression *vmp* cassette (see Fig S6 left panel and Table S3).”

L. 418-422. This statement is incorrect. B. burgdorferi vlsE sequence variations have been observed as early as 4 days post infection (see PMCID: PMC108403, doi: 10.1128/microbiolspec.MDNA3-0038-2014 and doi: 10.3389/fmicb.2024.1469411). Also, it would be better to state that the detection of organisms expressing the initial vlsE genotype is prolonged in immunocompromised mice, rather than saying that there is a “delay in switching”.

Answer: Thank you for the crucial correction and suggestion. Since the two articles you kindly suggested (doi: 10.1128/microbiolspec.MDNA3-0038-2014 and doi: 10.3389/fmicb.2024.1469411) were review papers, we have cited original research articles. (PMCID: PMC108403; Zhang JR et al.,1998, DOI: 10.1371/journal.ppat.1000293; Coutte L et al., 2009) for this phenomenon.

Lines 422-425: “In *B. burgdorferi*, a switch of expressing surface antigen gene *vlsE* has been observed in the early stages of infection (40,41). This is the first report demonstrating such a phenomenon in B. miyamotoi. The detection of organisms expressing the initial *vmp* genotype is prolonged in immunocompromised mice, suggesting that in immunocompetent animals, the expansion of an emerged new serotype population(s) occurs at the early stage of infection via immunological selection.”

Minor

L52. spirochetes

Thank you for the correction. We corrected.

L88. experimentally infected mice

Thank you for the correction. We corrected.

L96. Hermsii

Thank you for the correction. We corrected.

L123. of a C57BL/6J…

Thank you for the correction. We corrected.

L134. “relative deletion” rather than “gap”

Answer: Thank you for the comment. We modified throughout the manuscript.

L159 Arrowheads indicate

Thank you for the correction. We corrected.

L201 from any of the five clones

Thank you for the correction. We corrected.

L249 limiting dilution

Thank you for the correction. We corrected throughout the manuscript.

L321 mouse experiments*****

Thank you for the correction. We corrected.

Reviewer 2

L64-65: To the best of my knowledge, B. anserina is the only RF species which transmitted by Argas ticks. If you mention it, the louse-borne species B. recurrentis should be mentioned as well.

Answer: Thank you for the suggestion. We modified the sentence as follows.

Lines 64-65: “RF *Borrelia* are mainly transmitted by soft-bodied ticks of the genera *Ornithodoros* or *Argas*, with an exception of *B. recurrentis*, which is transmitted by lice.”

L77-78: It is important to differentiate between different mechanisms. Trypanosomes antigenic variations is similar to Borrelia AV with gene conversion being the main driver, whereases other pathogens use different mechanisms such as point mutations, reassortment and epigenetic changes.

Answer: Thank you for the suggestion. We modified the sentence as follows.

Lines 78-80: “It is known that several viruses, bacteria, fungi, and parasites that evade immune clearance by antigen switching via gene conversion, point mutations, reassortment and epigenetic changes (11-16).”

L110-112: Why is B. miyamotoi difficult to (re-)isolate from infected tissues? does this relate to low spirochetemia, fastidious growth requirements, or rapid antigenic shifts?

Answer: Thank you for the comment. The reasons why *B. miyamotoi* is difficult to cultivate in a medium are still unclear. However, we believe that the difficulty of re-isolating *B .miyamotoi* from mammalian samples is related to its low spirochetemia (there are more copies in ticks than in mice), its slow growth rate, its fastidious growth requirements and its low tolerance of environmental stress.

L120: which sequencing method was implemented?

Answer: Thank you for the comment. We used PacBio and Illumina sequencing methods for these two strains. We modified this sentence as follows.

Lines 121-123: “To determine the repertoires and genetic organizations of *vmp* gene cassettes, we sequenced two cloned Japanese strains, MYK1 clone G3 and M1-2Br clone H4 using the PacBio RS II system and Illumina MiSeq or HiSeq sequencer (Table S1) (3).”

L121-122: “None of the eight clones of MYK1, including clone G3, caused bacteremia when inoculated into laboratory mice (data not shown).”

What mouse strain was used? Inoculum conc.? Route of administration?

Answer: Thank you for the comments. We added the information in the text as follows.

Lines 124-126: “None of the eight clones of MYK1, including clone G3, caused bacteremia when 2 x 10^5^ bacteria were inoculated intraperitoneally into C57BL/6J (referred to as B6) mice.”

I wonder whether intradermal vs. intraperitoneal inoculation alters early antigenic switching kinetics.

Did this fact affect sequencing method or results?

Answer: Thank you for the thoughtful comments. Unfortunately, intradermal administration has not been performed, and it is unknown whether differences in the route of administration alters early antigenic switching kinetics or not. However, we have demonstrated that microbial levels in whole blood can be compared using another tick-borne pathogen, and that i.p. inoculation results in a higher copy number in whole blood than s.c. inoculation (unpublished data). Therefore, this issue should be addressed in future studies. If such a difference occurred, it may affect the results to some extent because of the lower concentration of DNA available.

L124-126: Did the infected mice exhibit any clinical signs?

Answer: Thank you for the comments. No apparent clinical signs were observed in the infected mice throughout the experimental period. We added the information in the text as follows.

Line 236: None of the mice exhibited any clinical signs in this study.

L127-133: read depth, assembly confidence around repetitive vmp regions and mapping coverage would strengthen claims of complete resolution.

Answer: Thank you for the comments. We added the details information of NGS in Table S1 and Table S5.

L132-137: The 4.6 kb gap on lp4 is noted. Gap closure strategies like long‐read polishing should be detailed to rule out assembly artifacts at the expression locus.

Answer: Thank you for the comments. In this study, we sequenced MYK1 G3 and M1-2Br H4 strains using PacBio and Illumina MiSeq or HiSeq technology. The 4.6 kb gap (relative deletion) in lp4 was detected by PacBio reads and it was confirmed by Southern blot analysis (see Figure S3). We have changed the description of “gap” to “relative deletion” to clarify this. In addition, we added a sentence as follows and detailed information of NGS in Table S1.

Lines 121-123: “we sequenced two cloned Japanese strains, MYK1 clone G3 and M1-2Br clone H4 using the PacBio RS II system and Illumina MiSeq or HiSeq sequencer (Table S1) (3).”

L141-142: What does “untypeable genes” mean? novel Vmp families or pseudogenes lacking hallmark motifs?

Answer: Thank you for the comments. The sequence of *vmp* have traditionally been classified into four types; however, some sequences described here was unable to be classified into any of these categories, so they were designated as untypeable genes. Most of them were presumably **pseudogenes lacking hallmark motifs**, and this assumption was further supported by the fact that no clones expressing these cassettes in our mouse experiments. To clarify this point, we have added a short description as follows.

Lines 145-147: “Through *in silico* analysis, we identified 44 *vmp* gene cassettes on the five linear plasmids in M1-2Br clone H4, which were classified into 22 *vlp*, 11 *vsp*, and 11 untypeable genes (most were presumably pseudogenes) (Fig 1).”

L220-232: Bacteremia dynamics are interpreted as evidence for antibody‐mediated clearance. However, no direct measurement of antibody titers is provided. Correlating serology with bacterial decline is essential to link immune pressure and switching kinetics.

Answer: Thank you for your comments. Our results support the importance of antibodies in bacterial elimination. To demonstrate the antibody titers, we have added a supplemental figure showing the results of the Western blot analysis (Figure S12). An increase in IgG levels was observed at 10 dpi, suggesting that IgG antibodies may be involved in the clearance of the bacteria. We added the following sentence in Discussion and provided the information on the method for Western blot analysis in the Materials and Methods section.

Lines 430-434: “The fact that *B. miyamotoi* was not eliminated in SCID mice but was no longer detected in immunocompetent mice after 10 dpi (Fig 2) highlights the importance of antibodies in bacterial elimination. Consistent with this, we also found that IgG levels increased in serum of mice at 10 dpi, as determined by Western blot analysis using whole-cell lysates of *B. miyamotoi* MYK2 clone A9 or MYK4 clone C8 (Fig S12).”

L291-292: Could this early conversion be classified as “spontaneous antigenic variation”, independent of adaptive immunity?

Answer: Thank you for the comments. It should be noted that the expression *vmp* cassette was not altered after several passages in BSK-M medium. This suggests that the phenomenon may be the result of some selection pressure from factors other than antibodies although spontaneous antigenic variation may occur at a very low frequency. We mention this in the Discussion as follows.

Lines 427-430: “Interestingly, the expression *vmp* cassette change was not observed in BSK-M medium but was observed in SCID mice, suggesting that factors other than antibodies may play a role in this phenomenon although spontaneous antigenic variation may occur at a very low frequency.”

L306-314: The hypothesis of long-segment conversion assumes homology‐driven recombination. However, the absence of DHS‐like motifs suggests alternative recombination mediators. The authors should search for associated recombination‐associated genes (encode resolvases or transposases) in proximity to vmp clusters.

Answer: Thank you for the suggestion. As you rightly pointed out, we are intensively exploring this. However, the genes potentially associated recombination based on the annotation-based search. Although some gene that encodes a protein of unknown function may be responsible, it is currently difficult to experimentally analyze it because genetic manipulation has not yet been developed for B. miyamotoi. We hope that we could identify the recombination‐associated gene(s) in future.

L432-436: “The most important finding of this study was probably the elucidation of the mechanism underlying the change in expression vmp cassette (antigenic variation) in B.miyamotoi”. Although this study provides a comprehensive analysis of expression locus segment conversion in B. miyamotoi, the exact mechanism and conversion drivers remain unidentified.

Answer: Thank you for this comment. We modified the discussion as follows.

Lines 442-446: “The most important finding of this study was the demonstration of dramatic changes in expression *vmp* cassette (antigenic variation) at an early stage of infection in *B. miyamotoi*, based on the genetic organization of *vmp* gene cassettes and the results of analyses of the segments from the expression cassette to the right end of the lp4 *vmp* expression plasmid (referred to as “expression locus segment” in this article) in reisolates obtained from infected mice.”

Regarding the comment that the exact mechanism and conversion drivers remain unidentified, we fully agree with reviewer as indicated on Lines 456-457 and Lines 452-464. We think that this is the most interesting and important scientific issue to be addressed in future.

Reviewer 3

1. Paragraph beginning with line 127; would be helpful to include the GenBank accession number here for the genome sequence of the strains. The accession numbers given in the supplementary tables were difficult to find on the NCBI search (at least for me).

Answer: Thank you for the comment. We modified as follows.

Lines 130-131: “By this sequencing, we obtained almost complete genome sequences of the two clones, MYK1 clone G3 (AP024375-AP024390) and M12Br clone H4 (AP024356-AP024370).”

The data are not publicly available at this stage, but they will be released upon acceptance. For your review, the file intended for public release is attached herewith.

2. Line 247; this was an area of confusion regarding which supplementary Tables to check. Suggest referencing Table S3 on line 248 instead of having both S2 and S3 on line 252. Also suggest a separate column on Table S3 stating the original expressed vmp gene.

Answer: Thank you for the suggestion. To clarify it, we have moved Table S2 to the next section and renamed as Table S4 (see Lines 250-258). And the expressed *vmp* cassette of original strains were indicated in Table S3.

3. Figure 5; suggest arrows to indicate the bands with the expression plasmid.

Answer: Thank you for the suggestion. Following the suggestion, we modified Figure 5 and its figure legend.

4. Lines 400-401; add the reference Hoornstra et al., iScience 2024. Genome sequences of 21 isolates were documented.

Answer: Thank you for the comment. We modified as suggested.

5. Line 464; Is there a reference that defines the ingredients of BSK-M? I’m not familiar with this media or how if differs from BSK-II, BSK-H, or BSK-R. If not, consider adding the specifics here.

Answer: Thank you for the comments. The composition of the BSK-M medium used in our lab was published in Takano et al., 2014 (DOI: 10.1371/journal.pone.0104532).

We have modified this sentence as follows and cited this paper as Reference 42.

Lines 474-478: “These *Borrelia* strains were grown at 30°C in modified Barbour–Stoenner–Kelly medium (BSK-M: using minimal essential medium alpha [Bio West, Germany]) supplemented with 10% rabbit serum under microaerophilic to anaerobic conditions, and the growth of spirochetes was examined using dark-field microscopy (at a magnification of 200×) (42).”

6. Line 522; “Two microliters of extracted DNA…”, replace microliters with micrograms to show quantity used.

Answer: Thank you for the comment. The average concentration of extracted DNA was 0.86 μg/μl. Since the DNA quantity was not measured for all samples, equal volumes (2 μl each) were used for qPCR, and calculations were based on the original volume used for DNA extraction in this study. We have added the sentence as follows

Lines 535-538: The average concentration of DNA extracted from whole blood was 0.86 μg/μl (measured by Qubit HR kit [Thermo Fisher Scientific, Waltham, MA, USA]). In this study, equal volumes of 2 μl were used for qPCR, and calculations were based on the original volume used for DNA extraction.
